# The metabolic syndrome modifies the mRNA expression profile of extracellular vesicles derived from porcine mesenchymal stem cells

**DOI:** 10.1186/s13098-018-0359-9

**Published:** 2018-07-21

**Authors:** Yu Meng, Alfonso Eirin, Xiang-Yang Zhu, Daniel R. O’Brien, Amir Lerman, Andre J. van Wijnen, Lilach O. Lerman

**Affiliations:** 1 Divisions of Nephrology and Hypertension, Mayo Clinic, 200 First Street SW, Rochester, MN 55905 USA; 2 Health Sciences Research & Biomedical Statistics and Informatics, Mayo Clinic, Rochester, MN USA; 3 Cardiovascular Diseases, Mayo Clinic, Rochester, MN USA; 4 Orthopedic Surgery, Mayo Clinic, Rochester, MN USA; 5 Department of Nephrology, The First Hospital Affiliated to Jinan University, Guangzhou, 510630 China

**Keywords:** Mesenchymal stem cells, Extracellular vesicles, mRNA, Metabolic syndrome, Swine

## Abstract

**Background:**

Mesenchymal stem cells (MSCs) perform paracrine functions by releasing extracellular vesicles (EVs) containing microRNA, mRNA, and proteins. We investigated the mRNA content of EVs in metabolic syndrome (MetS) and tested hypothesis that comorbidities interfere with the paracrine functionality of MSCs.

**Methods:**

Mesenchymal stem cells were collected from swine abdominal adipose tissue after 16 weeks of a low- (Lean) or high-calorie (MetS) diet (n = 5 each). We used next-generation mRNAs sequencing to identify mRNAs enriched and depleted in Lean- or MetS-EVs compared to the parent MSCs.

**Results:**

We found 88 and 130 mRNAs enriched in Lean-EVs and MetS-EVs, respectively, of which only eight were common genes encoding proteins related to the nucleus, endoplasmic reticulum, and membrane fraction. Lean-EVs were enriched with mRNAs primarily involved in transcription regulation and the transforming growth factor (TGF)-β signaling pathway, but devoid of genes related to regulation of inflammation. In contrast, MetS-EVs contained mRNAs involved in translational regulation and modulation of inflammation mediated by chemokines and cytokines, but lacked mRNAs related to TGF-β signaling. mRNAs enriched in EVs have the potential to target a significant proportion of genes enriched in EVs, but only 4% microRNA target genes overlap between Lean- and MetS-EVs. Co-culture with MetS-EVs also increased renal tubular cell inflammation in-vitro.

**Conclusions:**

Metabolic syndrome may affect immunomodulatory function of porcine MSCs by modifying mRNA profiles of the EVs that they produce and post-transcriptional regulation. These observations may have important implications for cell-based therapy, and support development of strategies to improve the efficacy of MSCs and their EVs.

**Electronic supplementary material:**

The online version of this article (10.1186/s13098-018-0359-9) contains supplementary material, which is available to authorized users.

## Background

Mesenchymal stem/stromal cells (MSCs) are non-embryonic stem cells that have the potential to proliferate and differentiate into several cell types, and can be isolated from the umbilical cord, bone marrow, adipose tissue, and other tissues [1]. Because MSCs possess important pro-angiogenic, immunomodulatory, and anti-inflammatory properties, they are a promising source for cell-based strategies that support tissue regeneration [2]. Indeed, over 200 clinical trials are currently testing the efficacy of MSC therapy for treating a variety of diseases [3].

MSCs exert their reparative actions in part by releasing extracellular vesicles (EVs), membrane microparticles including microvesicles and exosomes that shuttle their genetic and protein cargo to damaged tissues [4, 5]. Indeed, studies using a pig model of renal vascular disease have shown that EVs may activate an endogenous repair program in parenchymal cells [6, 7]. We have previously shown that adipose tissue-derived MSCs release EVs that transport gene and protein-based regulatory information to modulate angiogenesis, apoptosis, extracellular matrix remodeling and other cellular pathways in recipient cells [8–10], underscoring the reparative potential of MSCs. For example, MSC-derived EVs are selectively enriched with mRNAs encoding for transcription factors, Golgi apparatus components, and proteins involved in transforming growth factor (TGF)-β signaling. However, comorbidities and cardiovascular risk factors that alter the microenvironment from which these cells are harvested may potentially impact on the cargo packed within their EV progeny [11–13].

The metabolic syndrome (MetS) is a constellation of cardiovascular risk factors, including obesity, hypertension, dyslipidemia, increased serum triglycerides, and insulin resistance, that increases cardiovascular morbidity and mortality. In a recently developed porcine model of MetS and fat inflammation [14], we found that adipose tissue-derived MSCs show increased propensity for adipogenesis and for premature senescence compared to Lean-MSCs [15]. In the current study, we addressed the key question whether this phenotypic change is accompanied by altered packaging of mRNA cargo in their EV paracrine vectors. We tested the hypothesis that MetS affects the phenotype of MSCs, which in turn modifies the mRNA expression profile in EVs derived from MSCs. Expression profiles of mRNAs were obtained in EVs and their parent MSCs, using high-throughput RNA sequencing (RNA-seq). We show that the mRNA cargo of MetS-EVs is characterized by genes primarily involved in inflammation. Yet, mRNAs enriched in Lean-EVs are involved in tissue repair and TGF-β signaling, and these mRNAs appear to be excluded from MetS-EVs. Lastly, microRNAs enriched in EVs have the potential to target a significant proportion of genes enriched in Lean and MetS EVs, implying post-transcriptional regulatory mechanisms. These observations may help refine cell-based therapy and support development of adequate strategies to improve the efficacy of MSCs and their EVs.

## Methods

### Experimental design

Animals were studied with the approval of the Institutional Animal Care and Use Committee. Three-month-old female domestic pigs were randomized as MetS or Lean (n = 5 each). Lean pigs were fed with standard chow (13% protein, 2% fat, 6% fiber, Purina Animal Nutrition LLC, MN), and MetS pigs with a high-cholesterol/carbohydrate diet (5B4L, protein 16.1%, ether extract fat 43.0%, and carbohydrates 40.8%, Purina Test Diet, Richmond, IN) [14] for a total of 16 weeks. All animals had free access to water.

Body weight and blood pressure (using an intra-arterial catheter) were recorded after 16 weeks of diet. Total cholesterol, low-density lipoprotein (LDL), triglyceride, fasting glucose, and fasting insulin levels were measured by enzymatic methods. Insulin resistance was assessed by the homeostasis model assessment of insulin resistance (HOMA-IR). After completion of all studies, animals were euthanized with a lethal intravenous dose of sodium pentobarbital (100 mg/kg IV; Sleepaway^®^; Fort Dodge Laboratories, Fort Dodge, IA, https://www.zoetisus.com), and subcutaneous abdominal adipose tissue collected for MSC and EV isolation.

### MSC and EV isolation, characterization, and culture

Mesenchymal stem cells were isolated from swine subcutaneous abdominal fat tissue, which was digested in collagenase-H, filtered, and cultured for 3 weeks in advanced MEM medium (Gibco/Invitrogen) supplemented with 5% platelet lysate, as previously described [10]. At passage 3, MSCs were collected and cellular phenotype confirmed by the expression of the MSCs markers CD73, CD105, CD44, and CD90, as well as by their capacity for tri-lineage differentiation, as previously shown [16, 17].

Extracellular vesicles were isolated from passage 3-MSC supernatants (10 × 10^6^ cells) by ultracentrifugation, as previously described [8–10, 18, 19]. Samples were centrifuged (2000*g* for 20 min), and the supernatant collected and subsequently centrifuged (100,000*g* for 1 h) at 4 °C. EVs were collected, suspended in wash buffer medium 199, centrifuged once more (100,000*g* for 1 h), and characterized based on the expression of common EV (CD9 and CD63) and MSC surface markers, as previously shown [8, 10, 20].

### Sequencing of mRNA and data analysis

MSC and EV mRNA sequencing was performed as described [8, 18] and data analyzed using the MAP-RSeq v1.3 workflow [21, 22]. EdgeR2.6.2 [23] was used to analyze the differential expression to identify mRNAs enriched in Lean- and MetS-EVs compared to their parent MSCs. Expression values for each gene were normalized by the total number of reads per sample and corrected for gene length (reads per kilobasepair per million mapped reads, RPKM) (Additional File 1). Genes with RPKM > 0.1, fold-change (EVs/MSCs) > 1.4, and p < 0.05 (EVs vs. MSCs, Student’s t-test) were classified as enriched in EVs [24]. Conversely, we considered genes with RPKM > 0.1, fold-change (EVs/MSCs) < 0.7, and p < 0.05 to be excluded from EVs. Functional annotation clustering and pathway analysis were performed using DAVID 6.8 database (https://david-d.ncifcrf.gov/) and PANTHER Classification System (http://pantherdb.org/) [8, 10, 18].

To elucidate whether mRNAs enriched in MSC-derived EVs are implicated in insulin signaling, we used the GeneCards^®^ database (http://www.genecards.org/) to screen genes associated with insulin signaling [25].

### Validation of mRNA sequencing analysis

For validation, we randomly selected the mRNAs ANP32B, ARHGAP11B, CCL27, CLDN22, NR5A1, HIST1H2AJ, and PTX3, which were dysregulated in Lean and MetS-MSCs versus their daughter EVs, and measured their expression by quantitative polymerase chain reaction (qPCR) with GAPDH as reference gene. Total RNA was isolated from 5 × 10^5^ − 1 × 10^6^ MSC and EV samples. All primers were from ThermoFisher Scientific (Catalog Numbers: ANP32B: AP322J4, ARHGAP11B: APXGRAH, CCL27: ss03378478, CLDN22: ss04321456, NR5A1: ss03394295, HIST1H2AJ: AP2W7Y7, and PTX3: ss04328596).

### Interactions among microRNAs and mRNAs enriched in EVs

We have recently shown that Lean- and MetS-EVs were enriched in, respectively, 14 and 8 distinct microRNAs [26]. Hence, we sought to identify target genes for these microRNAs among the mRNAs packed within the same EVs. Venn diagrams were therefore constructed using VENNY 2.1 (http://bioinfogp.cnb.csic.es/tools/venny/) to visualize common miRNA target genes enriched in Lean- and MetS-EVs.

### MSC-derived EV effects on inflammation

Human monocytes were cultured for 18 h in RPMI 1640 media supplemented with M-CSF, LPS, and IF-γ to induce M1 polarization [27]. M1-polarized cells were cultured alone or co-cultured with Lean- and MetS-MSC-derived EVs (50 μg of EV protein). Expression of inducible nitric oxide synthase (iNOS) and arginase-1 (1:200, Santa Cruz, CA) was evaluated using Western blotting [17]. In addition, pig proximal kidney tubular epithelial cells (LLC-PK1, ATCC, Manassas) were cultured in Medium-199 (Gibco BRL, USA) containing 3% FBS [28] alone or co-cultured with Lean- and MetS-MSC-derived EVs (5 μg of EV protein). Tubular epithelial cell inflammation was evaluated by immunofluorescent staining with antibodies against tumor necrosis factor (TNF)-α (Santa Cruz, 1:200) and monocyte chemoattractant protein (MCP)-1 (MyBioSource, San Diego, CA, http://www.mybiosource.com 1:7500).

### Statistical analysis

Statistical analysis was performed using JMP 13.0 (SAS Institute, Cary, NC, USA). Data were expressed as mean ± standard deviation. Student’s t-test was used to evaluate significant differences between the Lean and MetS groups. Statistical significance was accepted if p ≤ 0.05.

## Results

### Systemic characteristics

The systemic characteristics of all animals at 16 weeks are shown in Table 1. Body weight and blood pressure were higher in MetS pigs compared to Lean. Fasting glucose levels were comparable, but other MetS indices like insulin level and HOMA-IR score were higher in MetS vs. Lean, as were total cholesterol, LDL, and triglyceride levels. These findings imply development of MetS in our pig model.

**Table 1:** Systemic characteristics in the experimental pig groups (n = 5 each) at 16 weeks

Parameter	Lean	MetS	p value
Body weight (kg)	69.3 ± 11.4	92.2 ± 4.4*	0.0005
Mean blood pressure (mmHg)	97.9 ± 13.1	124.1 ± 5.7*	0.002
Total cholesterol (mg/dL)	79.5 ± 7.3	354.2 ± 30.5*	< 0.0001
LDL cholesterol (mg/dL)	31.7 ± 6.9	349.0 ± 132.1*	0.0003
Triglycerides (mg/dL)	6.8 ± 1.7	17.1 ± 2.4*	0.0002
Fasting glucose (mg/dL)	108.8 ± 23.1	123.7 ± 22.3	0.207
Fasting insulin (µU/mL)	0.4 ± 0.1	0.7 ± 0.1*	0.002
HOMA-IR score	0.6 ± 0.1	1.7 ± 0.3*	< 0.0001

*HOMA* homeostasis model assessment of insulin resistance, *LDL* low-density lipoprotein, *Mets* metabolic syndrome

* p ≤ 0.05 vs. Lean

### Genes enriched in Lean- and MetS-EVs

**Fig. 1:**
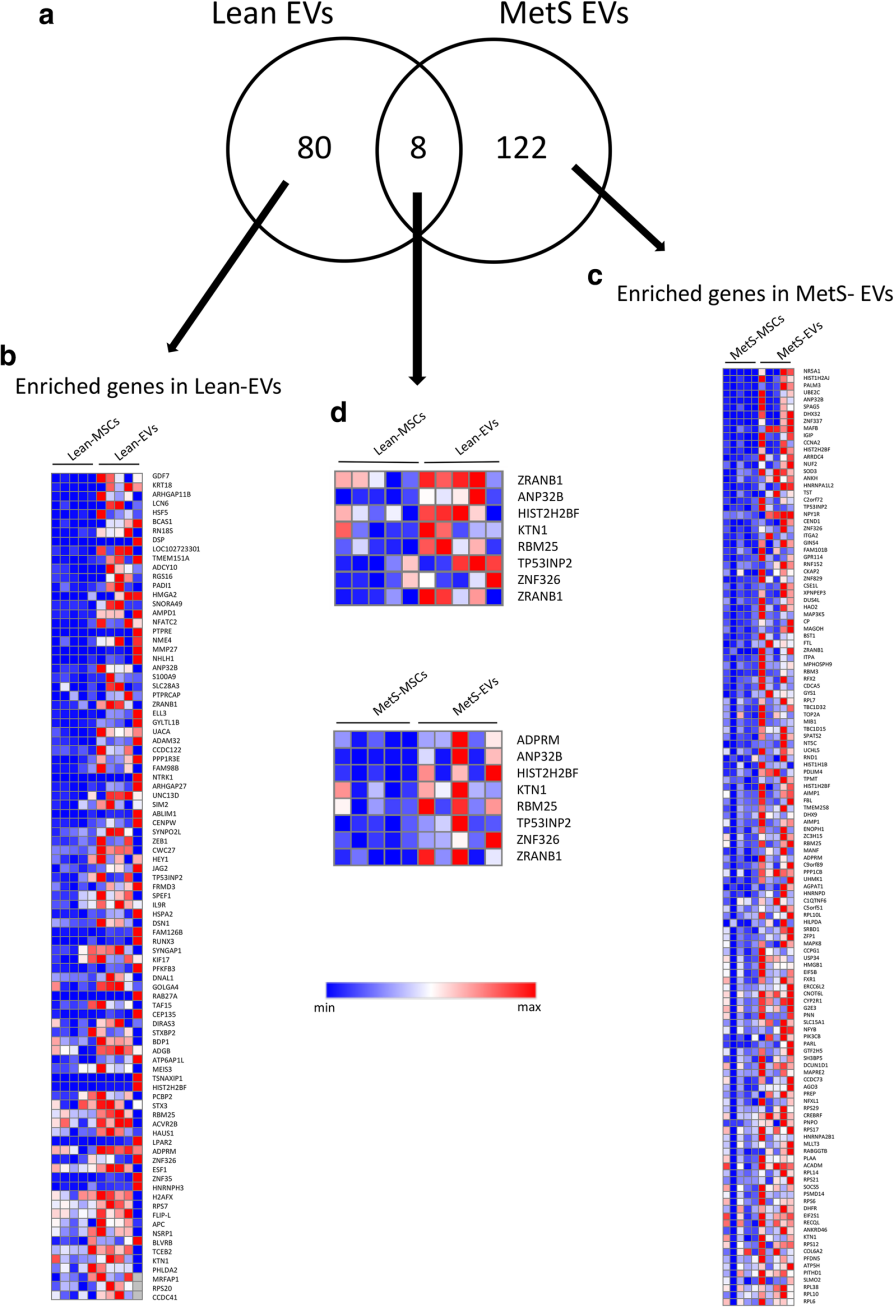
Enrichment of mRNAs in Lean- and MetS-EVs. **a** Of 16,656 annotated genes, mapping of RNA reads revealed 88 mRNAs enriched in Lean-EVs and 130 in MetS-EVs compared to their parent MSCs, with only eight common mRNAs. **b** Heat map of mRNAs enriched in Lean-EVs. **c** Heat map of mRNAs enriched in MetS-EVs. **d** Heat map of enriched genes shared between the Lean-EVs and MetS-EVs

Of all annotated genes (n = 16,656), mapping of RNA reads revealed 88 mRNAs upregulated in Lean-EVs compared to Lean-MSCs (Fig. 1a, b), and 130 mRNAs enriched in MetS-EVs compared to their parent MetS-MSCs (Fig. 1a, c). Of those, only eight enriched genes were shared between the Lean-EVs and MetS-EVs (Fig. 1a, d), and were found to encode proteins related to the nucleus, endoplasmic reticulum, and membrane fraction.

**Fig. 2:**
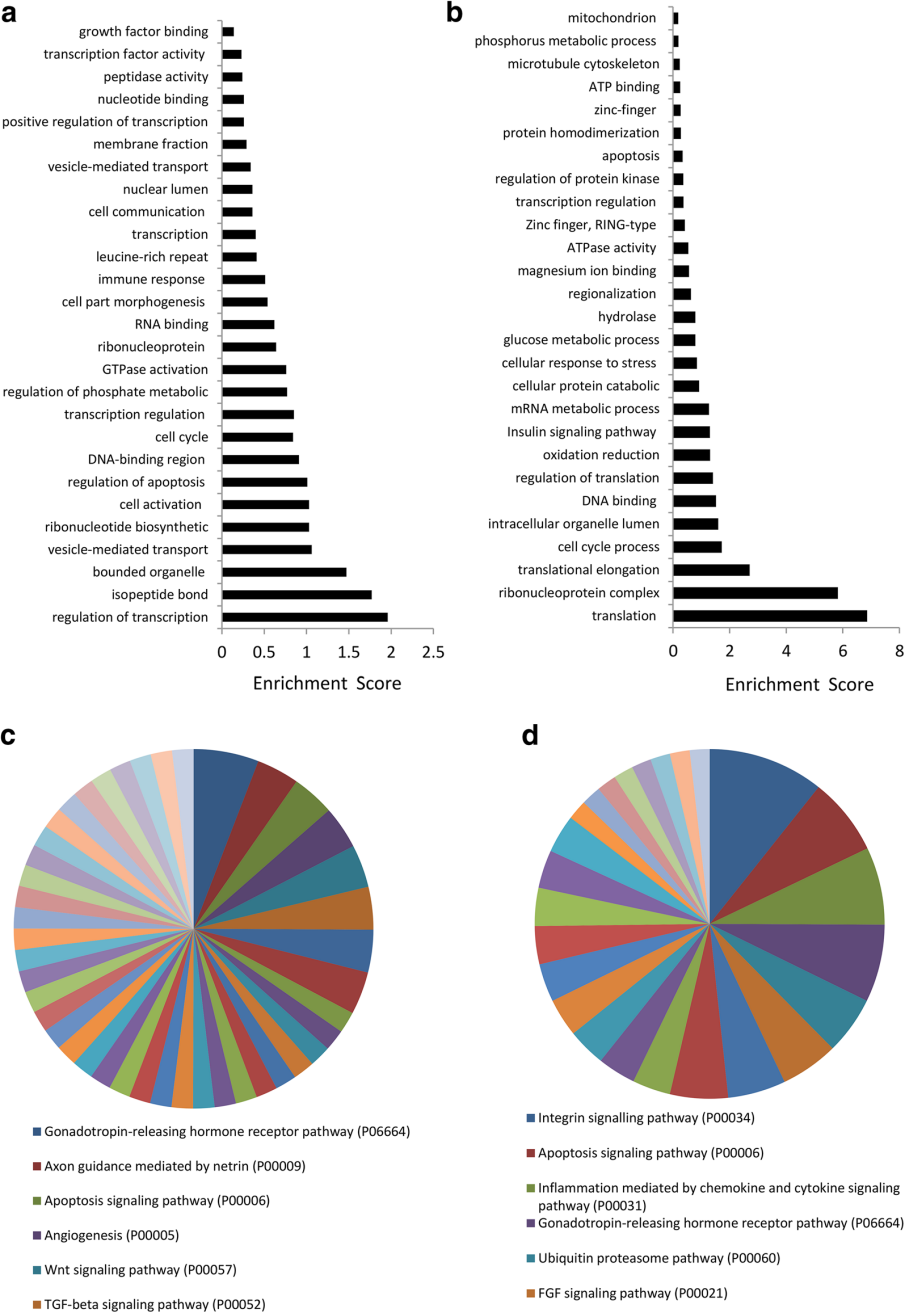
Functional pathway analysis of mRNAs enriched in Lean- and MetS-EVs. **a** Lean-EVs contained genes encoding regulators of transcription, isopeptide bond, non-membrane-bounded organelle, and vesicle-mediated transport, among others. **b** Genes enriched in MetS-EVs were primarily involved in translation elongation and ribonucleoprotein complex. **c** The top six functional categories of genes enriched in Lean-EVs showed pathways related to gonadotropin-releasing hormone receptor (P06664), axon guidance mediated by netrin (P00009), apoptosis signaling (P00006), angiogenesis (P00005), Wnt signaling (P00057), and TGF-β signaling (P00052). **d** The top six functional categories of genes enriched pathway in MetS-EVs included integrin signaling (P00034), apoptosis signaling (P00006), inflammation mediated by chemokine and cytokine signaling (P00031), ubiquitin proteasome pathway (P00060), and FGF signaling (P00021)

Functional annotation analysis showed that Lean-EVs selectively contain genes encoding regulators of transcription (Fig. 2a), including (Fig. 1b) meishomeobox-3 (MEIS3), hairy/enhancer-of-split related with YRPW motif 1 (HEY1), and heat-shock transcription factor family member 5 (HSF5). Biological pathway analysis showed that genes enriched in Lean-EVs encode proteins involved in 50 pathways. Overall, Lean-EVs enriched genes were involved in isopeptide bond, non-membrane-bounded organelle, and vesicle-mediated transport. Genes enriched in Lean-EVs also encode proteins involved in axon guidance mediated by netrin (P00009), angiogenesis (P00005), Wnt signaling pathway (P00057), and TGF-β signaling pathway (P00052) (Fig. 2c), confirming our previous observations in healthy pigs [8].

Contrarily, genes enriched in MetS-EVs encode proteins involved in translation elongation and ribonucleoprotein complex (Fig. 2b), including (Fig. 1c) eukaryotic translation initiation factor-2 subunit-alpha (EIF2S1), eukaryotic translation initiation factor 5B (EIF5B), ribosomal protein S17 (RPS17), and ribosomal protein (RP)-L6, 7, 10, 14. In contrast to Lean-EVs, mRNAs enriched in MetS-EVs are linked to 25 pathways and encode proteins involved in integrin signaling (P00034), inflammation mediated by chemokine and cytokine signaling (P00031), ubiquitin-proteasome (P00060), and fibroblast growth factor (FGF) signaling (P00021) pathways (Fig. 2d). Gonadotropin-releasing hormone receptor (P06664) and apoptosis signaling (P00006) were among the top six pathways regulated by genes enriched in both Lean- and MetS-EVs (Fig. 2c, d).

Twenty-one mRNAs related to insulin signaling were enriched in Lean-EVs (Table 2), whereas 19 distinct genes were enriched in MetS-EVs (Table 3). Two mRNAs enriched in MetS-EVs (NR5A1 and SLC15A1) were also enriched in MetS-MSCs, as we observed in a recent previous study [25].

**Table 2:** Insulin signaling-related mRNAs enriched in Lean-EVs

Official gene symbol	Gene name
KRT18	Keratin-18
DSP	Desmoplakin
ADCY10	Adenylate cyclase-10
RGS16	Regulator of G protein signaling-16
HMGA2	High mobility group AT-hook-2
AMPD1	Adenosine monophosphate deaminase-1
PTPRE	Protein tyrosine phosphatase, receptor type-E
S100A9	S100 calcium binding protein-A9
PPP1R3E	Protein phosphatase-1 regulatory subunit 3E
NTRK1	Neurotrophic receptor tyrosine kinase-1
HEY1	Hes related family BHLH transcription factor with YRPW Motif1
SYNGAP1	Synaptic Ras GTPase activating protein-1
PFKFB3	6-Phosphofructo-2-kinase/fructose-2,6-biphosphatase-3
RAB27A	RAB27A, member RAS oncogene family
TAF15	TATA-box binding protein associated factor-15
STXBP2	Syntaxin binding protein-2
PCBP2	Poly(RC) binding protein-2
STX3	Syntaxin-3
ACVR2B	Activin A receptor type 2B
H2AFX	H2A histone family member-X
BLVRB	Biliverdin reductase

**Table 3:** Insulin signaling-related mRNAs enriched in MetS-EVs

Official gene symbol	Gene name
NR5A1^a^	Nuclear receptor subfamily-5 group A member-1
MAFB	MAF BZIP transcription factor-B
CCNA2	Cyclin A2
SOD3	Superoxide dismutase-3
NPY1R	Neuropeptide Y receptor-Y1
ITGA2	Integrin subunit alpha-2
FTL	Ferritin light chain
HIST1H1B	Histone cluster-1 H1 family member-B
DHX9	DExH-box helicase-9
PPP1CB	Protein phosphatase-1 catalytic subunit beta
HNRNPD	Heterogeneous nuclear ribonucleoprotein-D
HMGB1	High mobility group box-1
EIF5B	Eukaryotic translation initiation factor-5B
SLC15A1^a^	Solute carrier family 15 member-1
PARL	Presenilin associated rhomboid like
PSMD14	Proteasome 26S subunit, non-ATPase-14
DHFR	Dihydrofolate reductase
EIF2S1	Eukaryotic translation initiation factor-2 subunit alpha
RECQL	RecQ like helicase

^a^ mRNAs also upregulated in MetS-MSCs compared to Lean-MSCs

### Genes excluded from Lean- and MetS-EVs

**Fig. 3:**
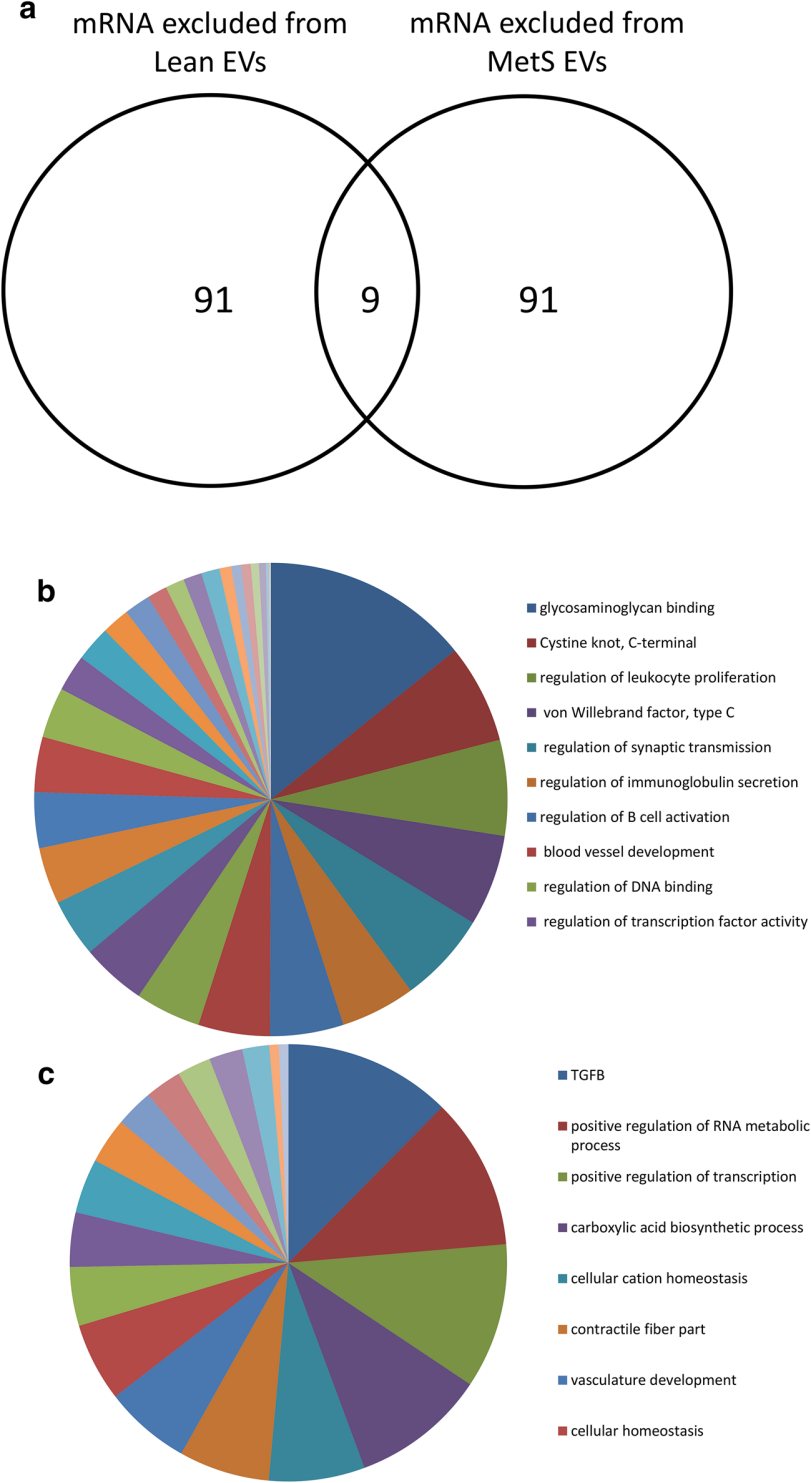
mRNAs excluded from Lean- and MetS-EVs. **a** Top 100 mRNAs excluded from Lean- and MetS- EVs revealed only nine common genes. Functional analysis of mRNAs showed selective genes excluded from Lean-EVs (**b**) and from MetS-EVs (**c**)

A total of 4166 mRNAs were excluded from Lean-EVs and 2449 from MetS-EVs. Annotation analysis of the top 100 mRNAs (filtered by fold-change EVs/MSCs) excluded from Lean- and MetS-EVs showed only nine common genes (Fig. 3a), which were involved in lipase activity, ion channel activity, oxidoreductase activity, and extracellular matrix structural constituents. Lean-EVs lacked inflammatory genes (23% of top 100 mRNAs excluded from Lean-EVs), such as those involved in regulation of leukocyte proliferation, immunoglobulin secretion, and B-cell activation (Fig. 3b). 12% of the top 100 mRNAs selectively excluded from MetS-EVs were related to TGF-β signaling, a pathway that seems to be blunted in MetS. MetS-EVs also lacked mRNAs related to positive regulation of RNA metabolic processes, positive regulation of transcription, carboxylic acid biosynthetic process, cellular cation homeostasis, and contractile fiber part (Fig. 3c).

### Validation of RNAseq findings

**Fig. 4:**
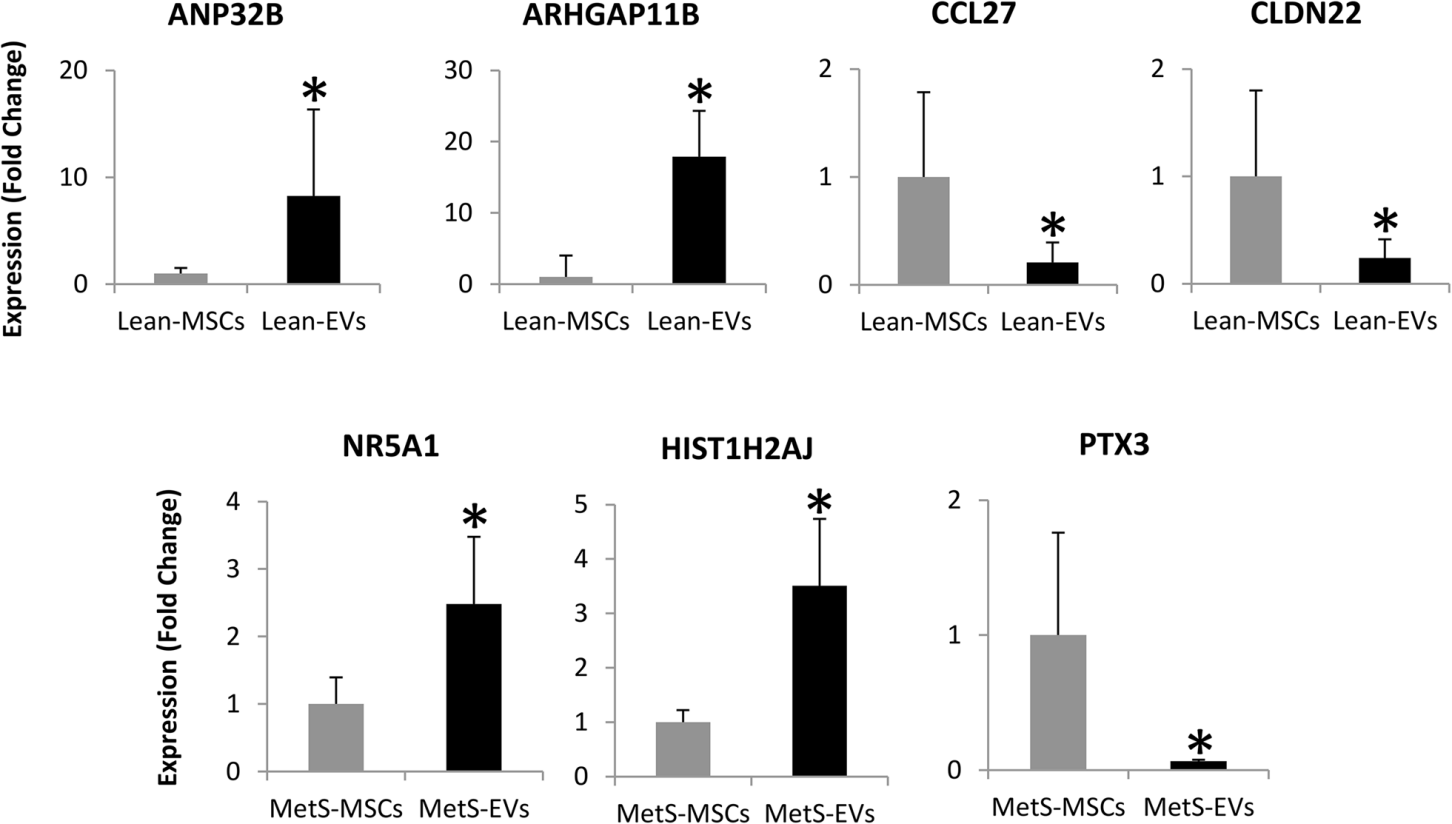
Expression (qPCR) of ANP32B, ARHGAP11B, CCL27, CLDN22, NR5A1, HIST1H2AJ, and PTX3 assessed by qPCR was concordant with the mRNA-seq findings. *p<0.05 vs. respective MSCs

PCR-determined expression of the random candidate mRNAs confirmed the same pattern detected by mRNA-seq. Specifically, ANP32B and ARHGAP11B were higher, and CCL27 and CLDN22 lower in Lean-EVs compared to their parent MSCs. Expression of NR5A1 and HIST1H2AJ was higher, but expression of PTX3 was lower in MetS-EVs vs. MetS-MSCs (Fig. 4).

### mRNA-microRNA interaction analysis

**Fig. 5:**
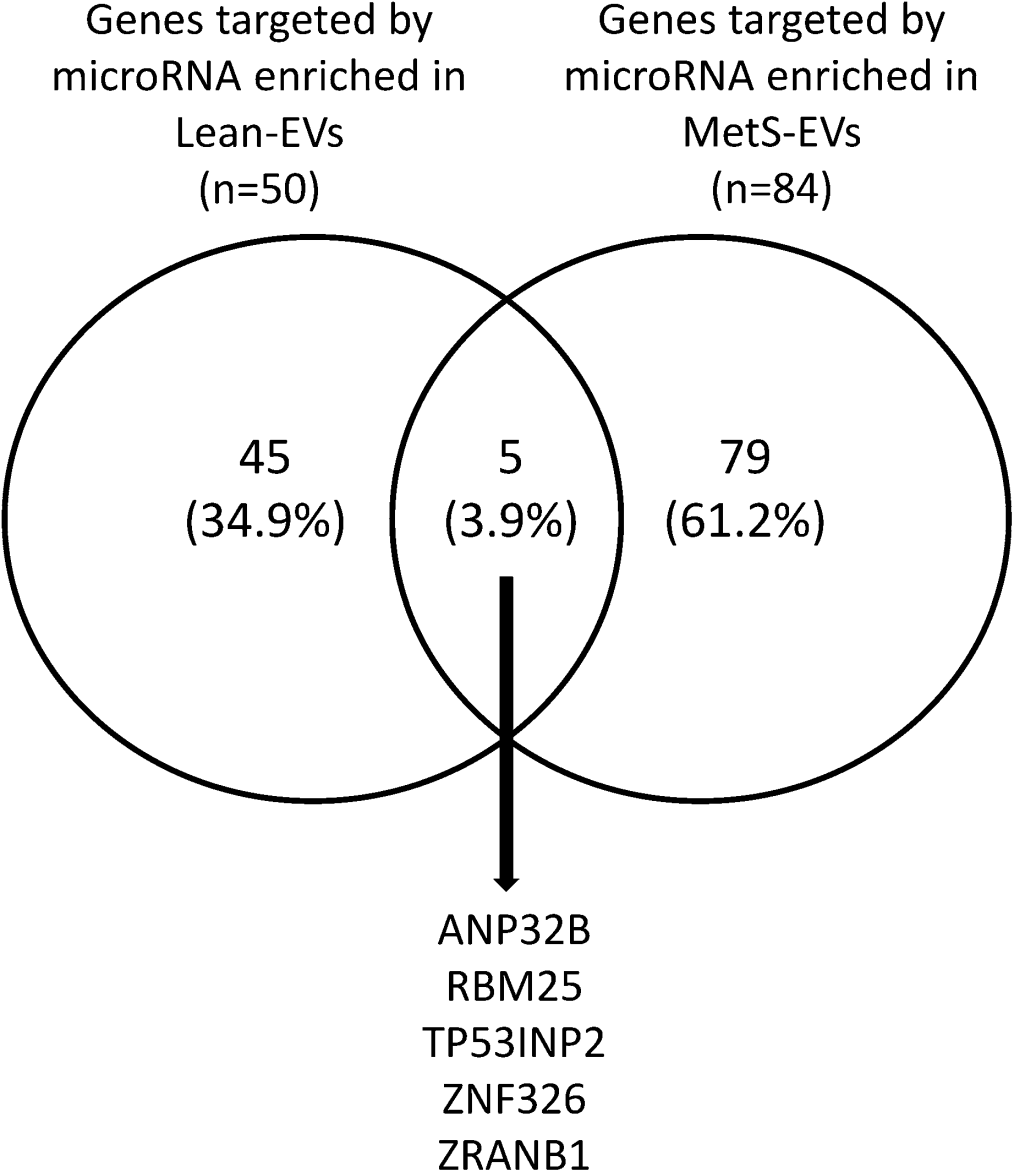
Interactions among microRNAs and mRNAs enriched in Lean and MetS EVs. microRNAs enriched in EVs have the potential to target a significant proportion of genes enriched in Lean and MetS-EVs (50 and 84, respectively), but only 5 (3.9%) of microRNA target genes overlap between Lean- and MetS-EVs

We took the opportunity to seek among the genes identified in the current study, targets for microRNA that we observed in a recent previous study. Interestingly, we found that those microRNAs enriched in EVs [18] have the potential to target a significant proportion of genes enriched in Lean and MetS-EVs (50 and 84, respectively), but only 5 (3.9%) of microRNA target genes overlap between Lean- and MetS-EVs (Fig. 5).

### Effects of MSC on inflammation

**Fig. 6:**
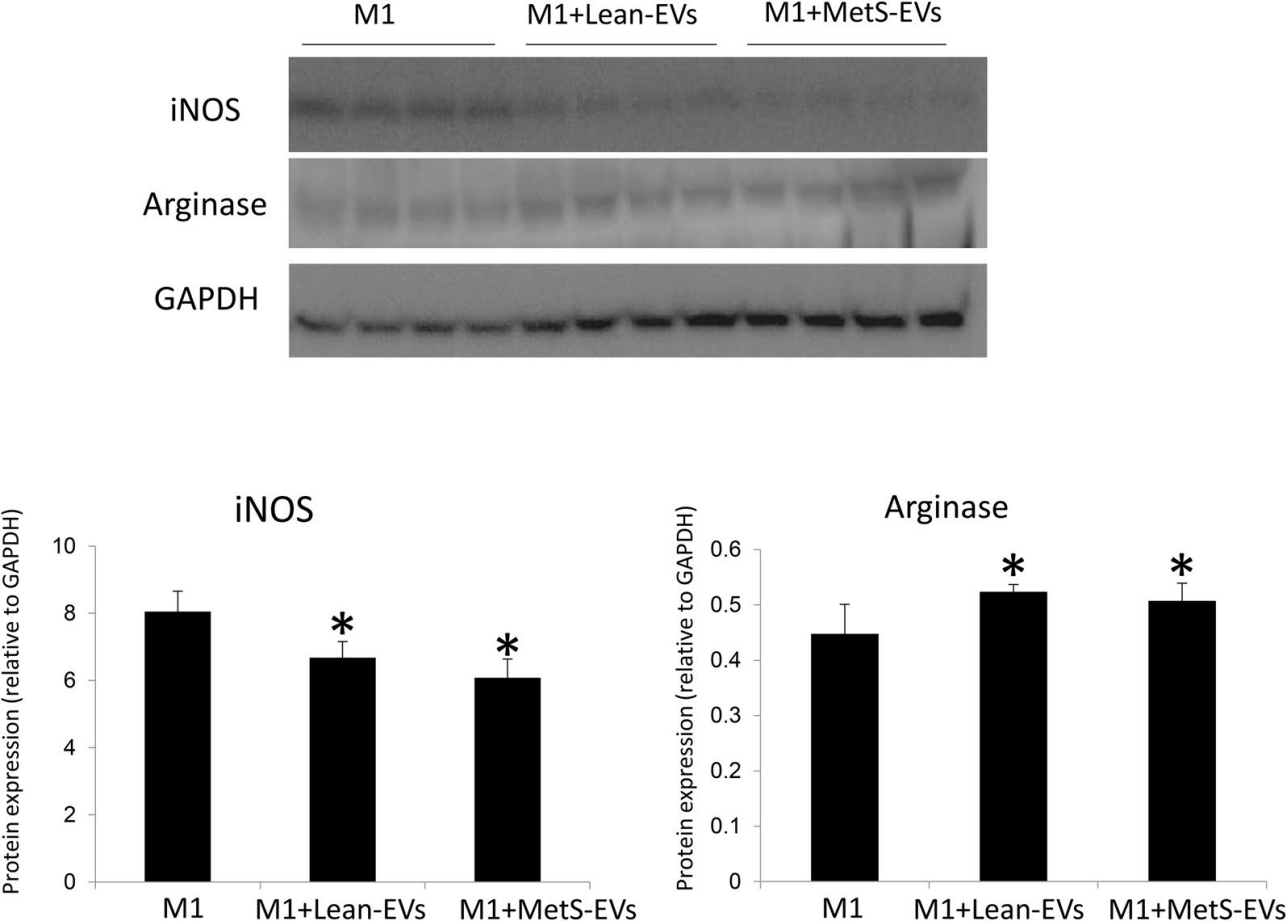
MSC-derived EVs effects on macrophages. Co-culture with either Lean- or MetS-MSC-derived EVs similarly increased the expression of inducible nitric oxide synthase (iNOS) and decreased the expression of arginase-1 compared to M1-polarized macrophages, suggesting a switch to M2 phenotype. *p<0.05 vs. M1

**Fig. 7:**
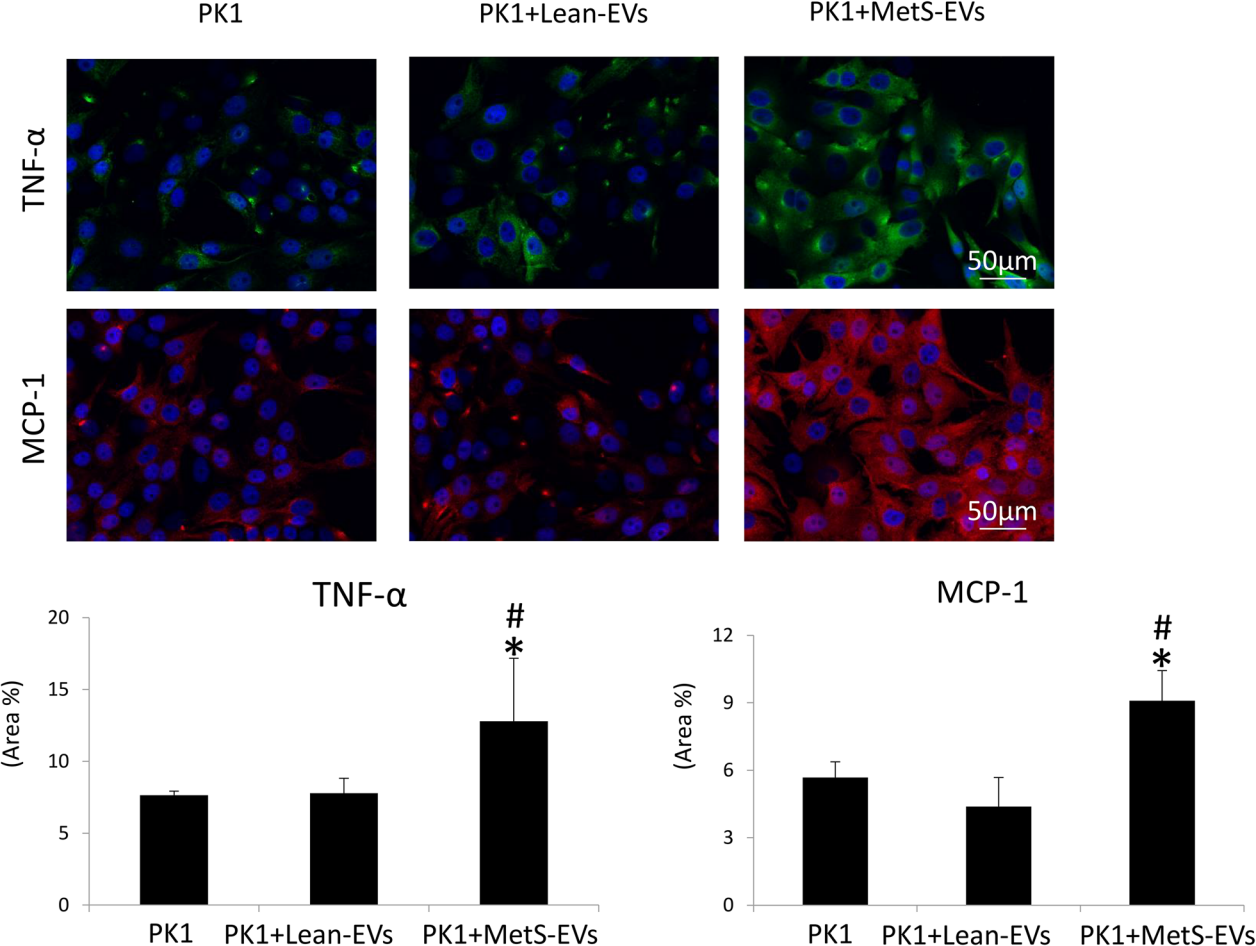
MSC-derived EVs effects on renal tubular cells. Expression of tumor necrosis factor (TNF)-α and monocyte chemoattractant protein (MCP)-1 was similar between in tubular cells (PK1) untreated of treated with Lean-EVs, but increased in those treated with MetS-EVs. *p<0.05 vs. PK1, ^#^p<0.05 vs. PK1 + Lean-EVs

Co-culture with either Lean- or MetS-MSC-derived EVs similarly decreased the expression of iNOS and increased the expression of arginase-1 compared to untreated M1-polarized macrophages (Fig. 6). However, when co-cultured with renal tubular cells, MetS-EVs induced a greater expression of TNF-α and MCP-1 compared to untreated cells and cells co-cultured with Lean-EVs (Fig. 7).

## Discussion

Our study employed high-throughput mRNA sequencing to interrogate mRNA expression in EVs and their parent MSCs, and explore the putative function of enriched or excluded genes in Lean- vs. MetS-EVs. The current study demonstrates that MetS modulates the mRNA cargo packed within porcine adipose tissue MSC-derived EVs, as the mRNA content of Lean- and MetS-EVs is substantially different. Lean-EVs are enriched with mRNAs primarily involved in transcription regulation and TGF-β signaling pathway, but not in genes related to regulation of inflammation. In contrast, MetS-EVs contain mRNAs involved in translational regulation and modulation of inflammation, but lack mRNAs related TGF-β signaling. Furthermore, we found that co-culture with MetS-EVs increases renal tubular cell inflammation in-vitro. Therefore, these observations demonstrate that MetS alters the genetic cargo of MSC-derived EVs, which might interfere with their ability to repair damaged tissues.

MSCs constitute a promising approach for cell therapy, but their function might be altered in a cardiovascular disease milieu, compromising MSC function. For example, MSCs from patients with MetS and diabetes show increased oxidative stress and autophagy, and fail to differentiate in functional adipocytes [11, 29]. Likewise, MSCs isolated from horses with MetS show impaired viability, oxidative stress, and senescence [12]. In agreement, we have previously shown that adipose tissue-derived MSCs obtained from pigs with MetS show enhanced adipogenic and osteogenic propensity and senescence compared to their Lean counterparts, which was partly mediated by adipose tissue inflammation [15]. More recently, Saleh et al. [30] summarized findings from preclinical studies testing the efficacy of adipose tissue-derived MSCs on obesity and MetS. They found promising beneficial effects of MSC transplantation on obesity and MetS, and conclude that paracrine effects of MSCs may constitute their most biologically significant role.

The paracrine function of MSCs is partly mediated by releasing EVs. As therapeutic tools, EVs may have a superior safety profile compared to their parent cells [31], because unlike cells they can be safely stored without losing function. In previous studies in healthy pigs [8–10], we found that adipose tissue-derived MSCs release EVs that transport genes and proteins capable of modulating several cellular pathways, particularly high levels of TGF-β-related genes and pro-angiogenic genes. The current study extends our previous findings, and underscores the selective content of genes encoding regulators of transcription, TGF-β signaling, and angiogenesis in Lean-EVs. TGF-β is an anti-inflammatory factor that regulates cell growth, differentiation, and fibrosis, as well as regulatory T-cells. Among its signaling partners are growth/differentiation factor-7 (or bone morphogenetic protein-12), which stimulates MSC differentiation [32] and induces tenogenesis in bone-marrow MSCs [33], and Activin receptor type-2B (ACVR2B), a member of bone morphogenetic protein signaling involved in regulating adult bone mass [34]. Wnt signaling is also one of the top functional categories of genes enriched in Lean-EVs, and has a close link to TGF-β signaling. They synergistically modulate embryonic development, fibrotic disease, and tumor progression through interactions among Smad, Axin, Dvl and β-catenin [35]. For example, Adenomatous Polyposis Coli (Apc), enriched in Lean-EVs (×1.5 vs. MSCs), is a Wnt pathway regulator that in conjunction with TGF-β regulates epithelial progenitor cell fate [36]. Hence, EVs may be endowed with functions linked to repair and regeneration of tissues and suppression of inflammation.

Compared to Lean-EVs, MetS EVs were found be enriched with mRNAs associated with inflammation, such as those encoding the integrin family proteins. Integrins are a family of transmembrane receptors that activate signal transduction pathways, inducing inflammation and early atherosclerosis. For example, ITGA2 (integrin alpha-2) triggers inflammation and endothelial dysfunction in patients with cardiovascular disease, chronic kidney disease, and type-2 diabetes [37–39], and joint inflammation in mouse models of rheumatoid arthritis [40]. Other pro-inflammatory genes enriched in MetS-EVs (Fig. 1c) included the phosphatidylinositol 4,5-bisphosphate 3-kinase catalytic subunit beta isoform (PIK3CB) [41] and several members of the ubiquitin-proteasome pathway involved in development of inflammatory and autoimmune diseases [42]. For example, the ubiquitin conjugating enzyme E2-C (UBE2C), which activates several other genes (SLC2A1-CCNB2-HMMR-KIF11-NUSAP1-PRC1-UBE2C), triggering inflammation [43], and the UCHL5 (or ubiquitin C-terminal hydrolase UCH37), which plays an important role in inflammation and host defense [44]. Lastly, mRNAs enriched in MetS-EVs encode proteins involved in FGF signaling, a family of proteins which can contribute to pathological conditions by modulating expression of inflammatory cytokines [45]. Hence, MetS-EVs have specific pro-inflammatory signatures that may impair the ability of MSCs to repair damaged tissues.

Genes that modulate apoptosis were similarly enriched in Lean- and MetS-EVs, but further analysis showed that those enriched in Lean-EVs encoded anti-apoptotic proteins, such as CFLAR (CASP8 and FADD-like apoptosis regulator) and heat-shock-related 70-kDa protein-2 (HSPA2). CFLARL-deficient T-cells undergo severe cell death upon T-cell receptor stimulation, involving apoptosis and necroptosis [46]. Likewise, HSPA2 increases long-term survival of cells subjected to heat shock and proteasome inhibitors, and can be a part of a system protecting cells against cytotoxic stimuli inducing proteotoxic stress [47]. In contrast, apoptosis-related genes that were enriched in MetS-EVs promote autophagy and apoptosis, including EIF2S1 and MAP3K5. EIF2S1 is an ER stress sensor that induces mitophagy through ER stress activation due to reactive oxygen species (ROS) accumulation, and MAP3K5 (also known as apoptosis signal-regulating kinase-1; ASK1) is a key component of ROS-induced JNK and p38 activation that activates apoptosis.

Our analysis also identified several mRNAs related to insulin signaling enriched in EVS, which were different between Lean-EVs and MetS-EVs. Interestingly, two mRNAs enriched in MetS-EVs were also enriched in MetS-MSCs, as we observed in a recent study [25]. The nuclear receptor subfamily-5 group-A member-1 (NR5A1, or steroidogenic factor-1), which modulates the expression of insulin-like factor 3, relaxin-like factor [48], and the oligopeptide transporter PepT1 solute carrier family-15 member-1 (SLC15A1), which is highly regulated by insulin [49]. These observations suggest that MetS alters mRNA expression related to insulin signaling in adipose tissue-derived MSCs and their daughter EVs.

To test whether MetS-induced changes in the cargo of MSC-derived EVs impact their anti-inflammatory and immunomodulatory potential, we compared the effects of Lean- and MetS-EVs in M1-polarized macrophages and renal tubular epithelial cells in vitro. We have previously demonstrated the ability of porcine adipose tissue-derived MSCs to switch macrophages from a pro-inflammatory M1 phenotype to M2, a repair-linked phenotype [17]. In the current study, we found that co-culture of M1-polarized macrophages with either Lean- or MetS-MSC-derived EVs similarly decreased the expression of the M1 markers and increased expression of M2 markers, in accordance with the similar capacity of Lean- and MetS-MSCs to restore an M2 phenotype in activated macrophages [15]. Contrarily, we found that expression of the pro-inflammatory cytokines TNF-α and MCP-1 was higher in renal tubular cells co-cultured with MetS-EVs compared to untreated cells and cells co-cultured with Lean-EVs, consistent with our previous observation that TNF-α magnified MSC dysfunction in MetS [15]. Taken together, these observations reflect the complex regulation of inflammatory responses, and suggest that MetS-MSCs and their EVs induce pro-inflammatory cytokines, but do not directly alter macrophage phenotype.

These observations have important clinical implications. Autologous transplantation is preferred for MSC therapy in patients, many of who have comorbidities and cardiovascular risk factors. The primary mechanism of action of MSCs is the delivery of EVs carrying mRNAs, microRNAs, and proteins that are transferred to damaged cells. Thus, our observation that MetS induces changes in the mRNA cargo of MSC-derived EVs that affects their immunomodulatory properties in vitro suggests that the reparative capacity of these cells may be limited in subjects with MetS.

Our study has a number of strengths, including a novel swine model of MetS, the comprehensive characterization of the mRNA cargo of Lean- and MetS-EVs, and in vitro studies in macrophages and renal tubular cells to elucidate the impact of MetS-induced changes in the cargo of EVs on their immunomodulatory function. On the other hand, our study is limited by the modest number of animals and short duration of MetS. Nevertheless, MetS pigs developed several features of human MetS, and we detected a clear difference in the mRNA content of their EVs compared to their Lean counterparts. The MSC mRNA profile can change with the passage of cells [50], thus we opted to study the 3rd passage cells that are relatively stable [51]. Additional studies are needed to explore the other functional ramification of the distinctive expression profile in MetS-EVs, and whether their mRNA content imposes a differential effect compared to Lean-EV on the phenotype of their recipient cells.

## Conclusions

Using next-generation sequencing analysis, we discovered a distinct profile of mRNAs expressed in Lean- and MetS-MSC-derived EVs. Genes enriched in Lean-EVs may reflect their predominant roles in regulation of transcription, TGF-β signaling, and angiogenesis-associated proteins. In contrast, mRNAs enriched in MetS-EVs are primarily responsible for encoding pro-inflammatory proteins, and selectively depleted of proteins known to be implicated in the TGF-β signaling pathway. Genes enriched in Lean-EVs encode proteins involved in transcriptional regulation, whereas those packed in MetS-EVs modulate translation. Lastly, co-culture with either MetS-EVs increases renal tubular cell inflammation in-vitro. These findings indicate that MetS modulates the genetic cargo and possibly functionality of EVs, vectors of MSC inter-cellular communication. Although our mRNA-microRNA analysis suggests important post-transcriptional regulation, the underlying mechanisms and ramification of these differences warrant further investigation. These observations have important implications for cell-based therapy and may support development of strategies to improve the efficacy of MSCs and their EVs.

## Additional file

**Additional file 1.** Individual mRNA expression levels obtained by RNAseq in Lean- and MetS-MSCs and their daughter EVs (Mvex) (n = 5 each). Expression values for each gene (Raw Counts) were normalized by the total number of reads per sample and corrected for gene length (reads per kilobasepair per million mapped reads, RPKM).

## Abbreviations

MSCs: mesenchymal stem cells
EVs: extracellular vesicles
TGF: transforming growth factor
MetS: metabolic syndrome
RNA-seq: RNA sequencing
LDL: low-density lipoprotein
HOMA-IR: homeostasis model assessment of insulin resistance
RPKM: reads per kilobasepair per million mapped reads
qPCR: quantitative polymerase chain reaction
MEIS3: meishomeobox-3
HEY1: hairy/enhancer-of-split related with YRPW motif-1
HSF5: heat-shock transcription factor family member-5
ACVR2B: Activin receptor type-2B
Apc: Adenomatous Polyposis Coli
EIF2S1: eukaryotic translation initiation factor-2 subunit-alpha
EIF5B: eukaryotic translation initiation factor 5B
RPS17: ribosomal protein S17
ITAG2: integrin alpha-2
PIK3CB: phosphatidylinositol 4,5-bisphosphate 3-kinase catalytic subunit beta isoform
UBE2C: ubiquitin conjugating enzyme E2-C
CFLAR: CASP8 and FADD-like apoptosis regulator
HSPA2: heat-shock-related 70-kDa protein-2
ROS: reactive oxygen species
ASK1: apoptosis signal-regulating kinase-1
NR5A1: nuclear receptor subfamily-5 group A member 1
SLC15A1: solute carrier family-15 member-1
